# Barriers to clinical cosmetic and laser dermatology research in the academic setting by source of funding: a systematic review

**DOI:** 10.1007/s00403-025-04241-8

**Published:** 2025-05-31

**Authors:** Bianca Y. Kang, Saranya P. Wyles, Yakir Levin, Rawaa Almukhtar, Stephanie R. Jackson Cullison, Jayne S. Joo, Sami K. Saikaly, Shalinie Mahadeo, Michael Ong, Sophia Salingaros, Maria Hordinsky, Sarah A. Ibrahim, Devina Mehta, Shoko Mori, Edit B. Olasz Harken, Diana Bolotin, Kira Minkis, Murad Alam

**Affiliations:** 1 Department of Dermatology, Mayo Clinic, Scottsdale, AZ USA; 2 Department of Dermatology, Mayo Clinic, Rochester, MN USA; 3 Center for Regenerative Biotherapeutics, Mayo Clinic, Rochester, MN USA; 4 Department of Dermatology and Wellman Center for Photomedicine, Massachusetts General Hospital, Harvard Medical School, Boston, MA USA; 5 Dermatology, Scripps Clinic, La Jolla, CA USA; 6 Department of Dermatology and Cutaneous Biology, Thomas Jefferson University, Philadelphia, PA USA; 7 Department of Dermatology, University of California Davis, Sacramento, CA USA; 8 Department of Dermatology, University of Florida, Gainesville, FL USA; 9 Department of Dermatology, Weill Cornell Medicine, New York, NY USA; 10 Department of Dermatology, University of Minnesota, Minneapolis, MN USA; 11 Department of Dermatology, University of Wisconsin, Madison, WI USA; 12 Department of Dermatology, Zucker School of Medicine at Hofstra/Northwell, New Hyde Park, NY USA; 13 Department of Dermatology, Medical College of Wisconsin, Milwaukee, WI USA; 14 Section of Dermatology, University of Chicago, Chicago, IL USA; 15 Department of Dermatology, Northwestern Feinberg School of Medicine, 676 N Saint Clair Street, Suite 1600, Chicago, IL 60611 USA

**Keywords:** Clinical research, Cosmetic, Laser, Dermatology, Academic, Funding

## Abstract

Clinical research is a cornerstone of academic dermatology, including research in cosmetic and laser procedures. However, numerous barriers exist to conducting clinical research in an academic setting as compared to private practice. The objective of this study was to describe the barriers to clinical research in cosmetic and laser dermatology in the academic setting under three common funding scenarios: (1) industry sponsored, (2) unfunded, investigator-initiated, and (3) publicly funded, investigator-initiated. A systematic review of the literature was conducted to identify 17 publications regarding funding of clinical dermatology research. Inductive content analysis was used to extract, categorize, and understand the barriers related to clinical dermatology research, specifically in cosmetic dermatology, based on the type of funding. An expert panel of 11 board-certified dermatologists who practice and conduct research in cosmetic and laser dermatology at academic institutions reviewed these barriers, interpreted each barrier’s implications for academic cosmetic and laser dermatology research, and proposed possible approaches to overcoming each. Nine barriers were identified for each funding scenario, and a total of 60 approaches for mitigating these were suggested. Most barriers to industry sponsored research were related either to institutional hurdles or industry preferences. The most cited barrier to unfunded, investigator-initiated research was limited protected academic time. The most frequently cited barriers to publicly funded, investigator-initiated research were grant availability and disproportionate awarding of grants based on investigator demographics. Proposed approaches for overcoming barriers included recruiting the help of trainees, streamlining administrative paperwork, fostering collaboration between industry and academic centers, providing financial incentives, seeking out mentorship from other faculty, and collaborating with other investigators, departments, and institutions.

## Introduction

Clinical research in cosmetic and laser dermatology research is important in understanding the safety and efficacy of various diagnostic and therapeutic modalities, as well as to discover and develop new treatments for patients of all skin types. Such research may be conducted either in an academic (i.e., university-affiliated) or non-academic (i.e., private practice) setting, and there are advantages and barriers specific to each setting. Benefits of clinical research in the academic setting may include increased collaboration across specialties, improved access to research support (e.g., academic biostatisticians, data analysts, animal laboratories, etc.), protected time for scholarly activity, and opportunity to work with trainees, including medical students, residents, and fellows [1]. Research has long been regarded as a pillar of academic medicine, and many dermatology residency and fellowship graduates who are specifically interested in conducting research choose to pursue careers in academia. However, there are also barriers to clinical research that are either unique to or more prominent in the academic setting. These barriers vary further based on the funding source. The objective of this study was to describe the barriers to cosmetic and laser dermatology clinical research in the academic setting under three common funding scenarios: (1) industry sponsored, (2) unfunded, investigator-initiated, and (3) publicly funded (e.g., from governmental institutions, charities, foundations, etc.), investigator-initiated.

## Materials and methods

### Expert panel

A panel of experts was convened to examine barriers to conducting cosmetic and laser dermatology research. This panel reviewed a diversity of literature (see below for search strategy and refinement) and provided expert opinion regarding the identified barriers in order to interpret each barrier’s implications for academic cosmetic and laser dermatology research, as well as propose possible approaches for overcoming each barrier. All panelists were (1) American Board of Medical Specialties (ABMS) board-certified dermatologists; (2) held faculty positions teaching cosmetic and laser dermatology to residents at Accreditation Council for Graduate Medical Education (ACGME)-accredited dermatology residency programs in the US; and (3) either members of the Association of Academic Cosmetic Dermatology (AACD) Research Committee or the AACD Board of Directors.

### Search strategy

A systematic review of the literature was conducted to identify publications pertaining to funding of clinical dermatology research. Search terms were developed with a medical librarian. Keywords were selected from the National Library of Medicine (NLM) MeSH (Medical Subject Headings) and included “dermatology,” “economics,” “funded,” “sponsor,” and “financial management” (Table 1). Search was conducted using MEDLINE from January 2000 to May 2023. Reference lists from included articles were also reviewed, and manual journal searching was performed, to identify additional relevant articles.

**Table 1:** Search terms used

#	Search term
1	(“economics”[MeSH Terms] OR “economics”[Subheading] OR “fund*”[ti] OR “sponsor*”[ti] OR “financial management”[MeSH Terms] OR “Societies, medical”[Mesh] OR “publications/economics”[mesh] OR “publications/statistics and numerical data”[mesh] OR “academic medical centers”[Mesh] OR “education, medical”[mesh]) AND (“Research”[Mesh] OR “Clinical trials as topic”[Mesh] OR “ethics, research”[Mesh] OR “research support as topic”[Mesh] OR “research design”[mesh])
2	(“dermatology”[MeSH] OR “dermatologie”[tiab] OR “dermatology”[tiab]) AND ((“economics”[MeSH Terms] OR “economics”[Subheading] OR “funding”[tiab] OR “financial management”[MeSH Terms] OR “financial management”[tiab] OR “funded”[tiab] OR “funds”[tiab] OR “fundings”[tiab] OR “sponsored”[tiab] OR “sponsor”[tiab] OR “funding source”[tiab]) AND (“Dermatologists/economics”[Mesh] OR “Dermatology/economics”[Majr] OR “Financial Statements”[Majr] OR “Research”[Mesh] OR “Clinical Trials as Topic”[Mesh] OR “Ethics, Research”[Mesh] OR “Research Support as Topic”[Mesh] OR “Research Design”[Mesh]))

### Selection process

Upon elimination of duplicate articles, two investigators (SM and SS) independently screened titles and abstracts for inclusion, then assessed full-text articles for eligibility, with two additional investigators (MO and BYK) resolving any conflicts. Included records were articles describing acquisition of funding for or barriers to clinical dermatology research in academic or non-academic settings. All article types were included, including both reviews and original research. Records were excluded if they were (1) the wrong medical specialty (i.e., any other than dermatology); (2) abstracts only; or (3) not available in the English language. Although some included studies focused on subspecialties outside of cosmetic and laser dermatology (e.g., complex medical dermatology or pediatric dermatology), many barriers identified in these articles, such as those related to ethical review, institutional costs, staffing, and access to research infrastructure, were considered applicable to cosmetic and laser research, and therefore included in our analysis.

### Data extraction and deductive content analysis

Data extraction and inductive content analysis were conducted by four investigators (SM, SS, MO, BYK), following a modified version of the structured approach described by Sadeghi-Bazargani and colleagues [2]. Briefly, the analysis was performed in five steps:

1. Familiarization with the data (i.e., identifying and extracting relevant barriers from the selected studies);
2. Searching for themes (i.e., organizing extracted barriers into potential thematic categories);
3. Formulating themes (i.e., developing a thematic structure to reflect the identified barriers);
4. Naming themes (i.e., defining and labeling themes with illustrative examples); and
5. Assessing reliability, which was done by having at least two investigators independently agree on coding assignments, with conflicts resolved by a fifth investigator (MA).

The analysis was inductive, based on descriptions of barriers found within the included articles. Themes were refined through discussion among the research team and reviewed by the expert panel to ensure relevance to cosmetic and laser dermatology. The final list of barriers was further organized by the expert panel into three funding scenarios: (1) industry sponsored, (2) unfunded, investigator-initiated, and (3) publicly funded, investigator-initiated.

## Results

### Study selection and characteristics

**Fig. 1:**
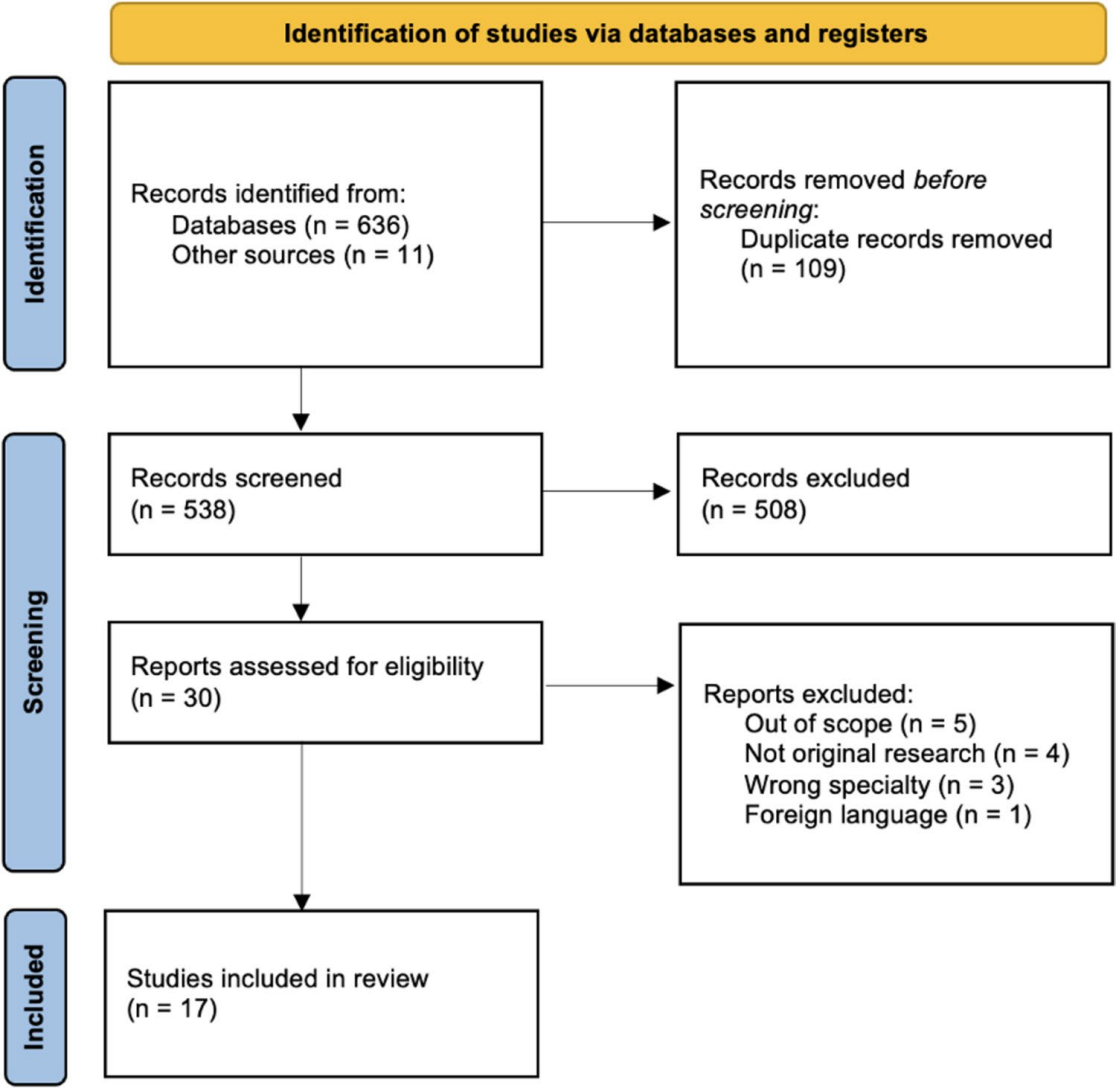
PRISMA flow diagram

After duplicates were removed, 538 records were screened by title and abstract, and 30 full-text articles were assessed for eligibility. Seventeen articles met inclusion criteria (Figure 1, Tables 1 and 2).

**Table 2:** Results of the systematic review

Article	Article type	Objective	Relevant funding scenario(s) and barriers identified	Additional key points
Prendergast et al. [3]	Review	Propose standards for conducting burden-of-illness and outcomes research studies involving collaborations between industry and academia	Industry sponsored ● COI	● Financial relationships between industry and academia foster research. ● Study design quality and execution are just as important as COI. ● Proposed standards (focusing on high-quality research with uniform standards; acknowledging and managing financial relationships; and promoting research transparency without restricting investigator behavior) promote productive relationships between industry, investigators, and academic institutions.
Leavitt et al. [4]	Bibliometric analysis	Evaluate the frequency of financial disclosures, and how often these correlate with true COI, in the plastic surgery, dermatology, and orthopedic literature	Industry sponsored ● Accrediting bodies ● COI Publicly funded, investigator-initiated ● Availability of grants	● The overall incidence of financial disclosures was low (8.8%), as was the incidence of true COI (5.5% across all three specialties, 2.2% in dermatology). ● The peer-review process is likely effective at eliminating biased and flawed studies with true COI.
Minkis et al. [5]	Review	Describe needs and potential corrective initiatives in resident education, patient experience, and clinical research within academic cosmetic dermatology	All ● Ethical review Industry sponsored ● Patient complexity	● Academic dermatologists may benefit from support in obtaining ethical approval and other time-consuming administrative tasks. ● The academic setting provides ample opportunity to study applications of cosmetic and laser procedures in treating patients with complex medical dermatologic conditions.
Schlafly and Sebro [6]	Cross-sectional study	Evaluate trends in how NIH funding was awarded by medical specialty from 2011 to 2020	Publicly funded, investigator-initiated ● Availability of grants ● Institutional prestige ● Investigator demographics	● NIH funding varies between medical specialties, which may affect research progress, investigator careers, and patient outcomes. ● Dermatology ranked 11 out of 19 specialties in average dollars of funding per active physician per year with $6.5k in funding per active physician per year, on average (range $0.1k - $47.8k). ● Early-career investigators typically receive smaller grants than more seasoned investigators.
Waldman et al. [7]	Review	Identify current gaps in the practice of cosmetic dermatology and cosmetics education, and how to best overcome these limitations	Industry sponsored ● Comparative efficacy ● Patient complexity Unfunded, investigator-initiated ● Collaboration with other researchers ● Trainee participation Publicly funded, investigator-initiated ● Availability of grants	● Because cosmetic procedures are not reimbursed, few payers outside of industry are likely to invest in cosmetic and laser dermatology research ● Comparative efficacy research is difficult due to lack of standardized outcomes and measures, and due to industry preference to avoid studies comparing their efficacy against competitors’.
Alam et al. [8]	Review	Identify current practice in laser dermatology, gaps in practice, and recommendations for improvement	Industry sponsored ● Comparative efficacy ● Device safety Unfunded, investigator-initiated ● Staffing ● Device cost Publicly funded, investigator-initiated ● Dermatology subspecialty ● Device cost	● Compared to cosmetic dermatology, laser research has more variables to consider and control for, including spot size, fluence, pulse duration, and cooling.
Hogan et al. [9]	Bibliometric analysis	Identify the practice settings of the most published and highly-cited authors of *Dermatologic Surgery* research	Industry sponsored ● Institutional bureaucracy Unfunded, investigator-initiated ● Protected time ● Staffing Publicly funded, investigator-initiated ● Protected time	● The majority of the most published authors in *Dermatologic Surgery* worked in private practice, though most also maintained academic appointments. ● Nearly half of the most highly-cited articles came from private practice dermatologists, and just over one-third were collaborative studies between private practice, academia, and/or industry.
Sugarman et al. [10]	Survey	Identify constraints limiting research activities among members of the Society of Pediatric Dermatology	Unfunded, investigator-initiated ● Collaboration with other researchers ● Protected time ● Statistical support and research infrastructure ● Training and mentorship Publicly funded, investigator-initiated ● Availability of grants ● Protected time ● Training and mentorship	● Of the 70 surveyed pediatric dermatologists, 98.5% reported perceived barriers to research productivity. The majority of these barriers were related to time (89.9%) or funding (71%) constraints. ● Manuscript provides a list of potential initiatives to increase research productivity.
Hartman et al. [11]	Review	Describe barriers and possible solutions to sustained research productivity throughout dermatologists’ careers	Industry sponsored ● Institutional bureaucracy ● Investigator compensation Unfunded, investigator-initiated ● Training and mentorship ● Trainee participation Publicly funded, investigator-initiated ● Availability of grants ● Training and mentorship	● Trivialization of skin disorders may be a culprit in the gap between disease burden and research funding. ● Medical students applying into dermatology are often more engaged in research than those applying to other specialties, but involvement in research is not sustained. ● Mentorship is associated with increased research productivity in terms of number of publications and amount of funding.
Mital et al. [12]	Cross-sectional study	Understand the factors influencing successful NIH funding at dermatology residency programs in 2014	Publicly funded, investigator-initiated ● Institutional prestige	● Compared to dermatology divisions, residency programs with departmental status were 3.86 times more likely to successfully receive NIH research funding. ● Higher quartile rankings for each dermatology program’s associated medical school, as well as location in the Western US and increased number of publications, faculty size, and faculty representation at conferences were also associated with increased funding.
Price et al. [13]	Cross-sectional study	Examine NIH funding trends in dermatology by investigator degree, gender, location and institution, from 2015 to 2019	Publicly funded, investigator-initiated ● Dermatology subspecialty ● Investigator demographics ● Institutional prestige	● There was a significant gender gap with male investigators receiving a larger portion of all grants and more funding per recipient than female investigators, and this persisted for all 5 years of the study. ● PhDs received the most funding, followed by MDs then MD/PhDs. ● The Northeast US received the majority of funding. ● More prestigious institutions received more funding.
Cheng et al. [14]	Cross-sectional study	Examine NIH funding trends in dermatology by investigator degree and gender, from 2009 to 2014	Publicly funded, investigator-initiated ● Investigator demographics ● Institutional prestige	● There was an overall downward trend in dermatologic research funding, particularly for investigators who were female (vs male) or who had an MD (vs PhD and MD/PhD).
Werth and Hordinsky [15]	Review	Describe approaches to increasing physician-directed research in complex medical dermatology	Unfunded, investigator-initiated ● Protected time ● Training and mentorship Publicly funded, investigator-initiated ● Protected time ● Medical specialty ● Training and mentorship	● Protected academic time and salary support are important to promote sustained research in complex medical dermatology. ● Collaboration with better funded medical specialties (e.g., internal medicine) can improve research productivity and understanding of complex diseases, but takes significant time.
Ali et al. [16]	Review	Describe virtual clinical trials and their implications in dermatologic research	Industry sponsored ● Cost of research	● Compared to traditional clinical trials, virtual clinical trials have improved cost-efficacy, higher recruitment rates, and lower drop-out rates. Virtual clinical trials are also conducted faster than traditional clinical trials. ● Challenges of virtual clinical trials include technological issues, selection bias for internet-savvy patients, and privacy concerns.
Dunst et al. [17]	Bibliometric analysis	Analyze the three top ranking dermatology journal for scientific content, author characteristics, and funding	Unfunded, investigator-initiated ● Collaboration with other researchers ● Dermatology subspecialty Publicly funded, investigator-initiated ● Dermatology subspecialty	● Cosmetic dermatology represented a minority (2.5%) of published articles, possibly due to the presence of strong subspecialty journals. ● Most (70%) publications were university-based. ● Half (51%) of published articles were funded, with grants (e.g., from governmental institutions, charities, and foundations) representing 67% of funding sources and pharmaceutical companies representing 33%.
Molina-Leyva et al. [18]	Bibliometric analysis	Analyze the resources and activities that are associated with higher scientific productivity at residency programs in Spain	Unfunded, investigator-initiated ● Protected time ● Staffing ● Trainee participation Publicly funded, investigator-initiated ● Protected time ● Investigator demographics	● More residents, research personnel, PhD dissertations, industry and publicly funded studies, as well as lower clinical workload were associated with higher scientific productivity.
Yaghoubnejad et al. [19]	Cross-sectional study	Examine NIH funding trends in dermatology by investigator number of publications, H-index, leadership positions in dermatological societies, gender, academic rank, and degree in 2014	Publicly funded, investigator-initiated ● Investigator demographics	● Number of publications, leadership dermatology societies, and H-index were correlated with NIH funding. ● Compared to other similar analyses, this study did not find a statistically significant association between NIH funding and academic rank gender, or degree.

*COI* conflict of interest, *NIH* National Institutes of Health

Review articles were the most common (7/17, 41.2%), followed by cross-sectional studies (5/17, 29.4%) and bibliometric analyses (4/17, 23.5%). There was one survey study (1/17, 5.9%). Three articles (17.6%) focused on cosmetic and laser dermatology. The remainder focused on general dermatology (9/17, 52.9%); multiple specialties, including dermatology (2/17, 11.8%); dermatologic surgery (1/17, 5.9%); pediatric dermatology (1/17, 5.9%); and complex medical dermatology (1/17, 5.9%).

Most articles (10/17, 58.8%) discussed more than one type of funding, typically for support of clinical trials. The most common type of funding discussed was public funding (15/17, 88.2%). Unfunded research (9/17, 52.9%) and industry sponsored research (8/17, 47.1%) were discussed in about half of included articles.

### Expert panel

The expert panel was composed of 11 dermatologists, each at a unique academic institution^1^. All panelists had experience conducting clinical research in cosmetic and laser dermatology in the academic setting.

### Industry sponsored clinical research

**Fig. 2:**
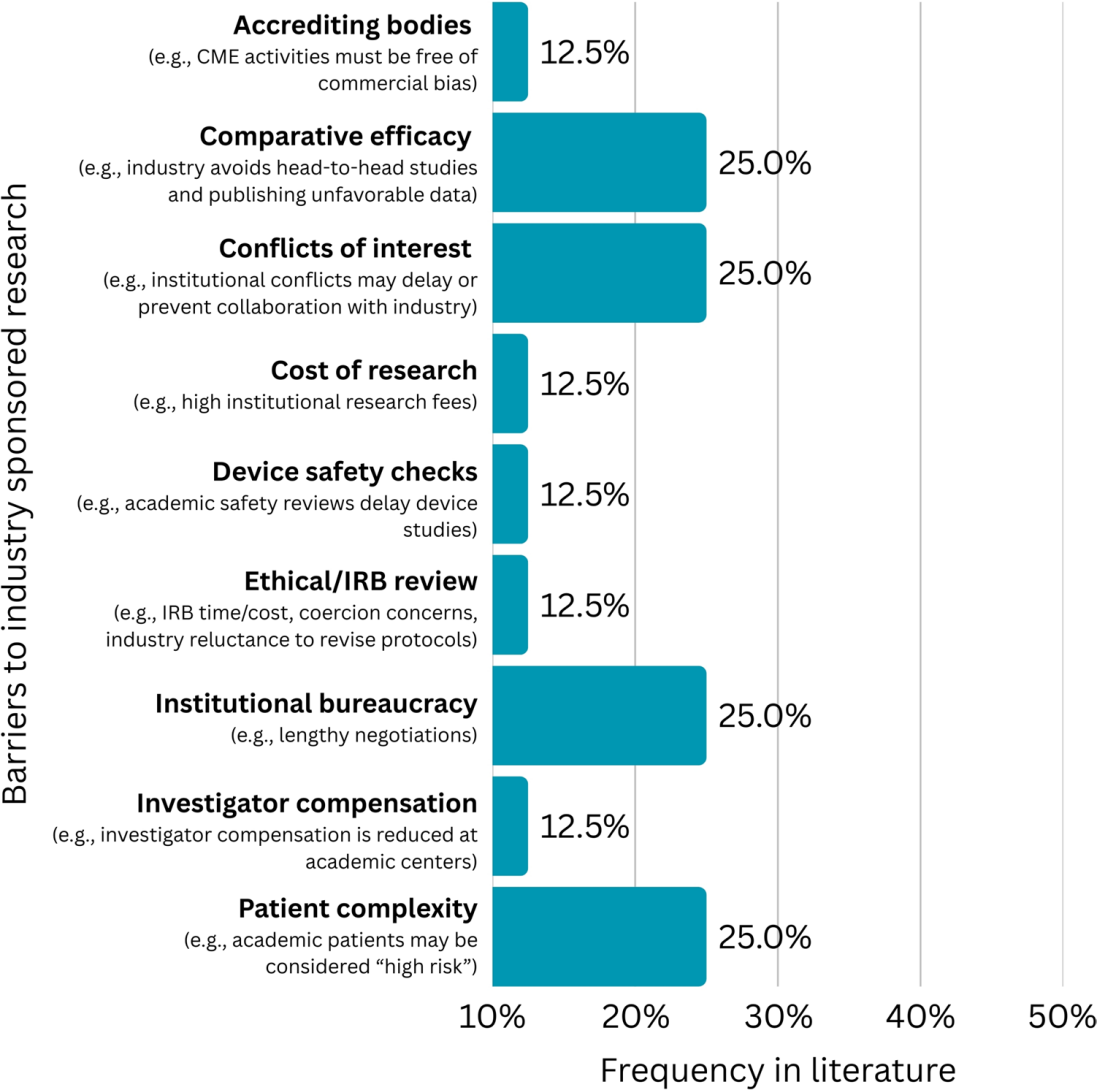
Frequency of barriers to conducting industry sponsored clinical research in cosmetic and laser dermatology in the academic setting, displayed as percentage of total identified articles pertaining to industry sponsored research (N = 8)

Nine barriers to industry sponsored clinical research in cosmetic and laser dermatology in the academic setting were identified, and the expert panel proposed a total of 20 possible approaches to overcoming these barriers (Figure 2, Table 3). Broadly, barriers were related to one of two factors: (1) institutional hurdles (e.g., concerns regarding conflicts of interest, institutional bureaucracy, ethical review, etc.) or (2) industry preferences (e.g., to avoid studies involving comparative efficacy or patients with complex disease).

**Table 3:** Barriers to conducting industry sponsored clinical research in cosmetic and laser dermatology in the academic setting, including description and proposed possible approaches to overcoming each barrier

Barrier	Description	Possible approaches to overcome each barrier
Accrediting bodies	ACGME requires educational activities to be free of commercial bias, which limits the exchange of novel research advances [4]	• Preference for investigator-initiated studies that are developed by researchers and presented to industry for funding • Explicit disclaimers that acknowledge that industry sponsors cannot suppress publication of results
Comparative efficacy	Industry may prefer to avoid studies comparing efficacy of their product or device against competitors’, and may prohibit publication of unfavorable data [7, 8]	• Studies may need to use a placebo or sham treatment comparator • Industry funding may be used for studies elucidating mechanism of action, safety, or utility in special populations (e.g., skin of color), instead of effectiveness
Conflicts of interest	Conflict with the scientific and financial interests of the academic institution may delay and even preclude collaboration with industry [3, 4]	• Implementation of conduct standards for relationships between industry and academia [3] • Increase collaboration between leadership in academic centers and industry through partnership facilitated by professional societies ex: Association of Academic Cosmetic Dermatologists (AACD), American Academy of Dermatology (AAD), American Society of Dermatologic Surgery (ASDS), etc.
Cost of research	Conducting research at academic institutions is costly (e.g., due to facility fees if the clinic is attached to a hospital) [16]	• Virtual or hybrid clinical trials (e.g., use web-based surveys, apps, etc.) [16] • Leverage advantages of performing trial in academic center (high volume patient base, diverse population, potential for multi-specialty collaboration, ability to perform additional testing: histopathologic, genetic, molecular testing, etc.)
Device safety checks	Lasers and other devices must go through time consuming regulatory and safety checks by the institution, which typically know little about these devices [8]	• Consider performing research on devices that are already installed and in operation • Dermatologist engagement in safety committees at their institution
Ethical/IRB review	IRB time and cost; IRB concern that free cosmetic treatments may be coercive; industry reluctance to revise protocols to comply with institution IRB recommendations [5]	• Cost for ethical approval may be mitigated by internal grants available at the institutional or departmental level • Direct communication with ethical review board analysts may be helpful to anticipate barriers and speed approval • Departments can hire research personnel to assist with ethical review board submission and revision
Institutional bureaucracy	Regulations at academic institutions require extensive negotiation prior to initiation of research [9, 10]	• Achieving departmental status and increasing faculty size [12] • Advocating to institutional leadership that funding from industry improves departmental research output [18]
Investigator compensation	Investigator compensation for participation in clinical trials may be significantly reduced at academic institutions compared to private practice, removing some incentive to get involved [11]	• Investigators may benefit in terms of academic prestige and advancement from senior authorship on significant research sponsored by industry • Industry may consider donations to faculty or investigator research accounts, departmental initiatives, or to resident training funds • Department leadership may incentivize clinical research productivity by considering this as a factor in physician compensation
Patient complexity	Industry, particularly companies focused on cosmetics and lasers, may not prioritize studying “high risk” patients (e.g., with connective tissue disease or other complex medical dermatologic conditions) [5, 7]	• Convey to industry the reputational benefits of being perceived as being concerned about the most vulnerable patients • Convey to industry the possible commercial success of providing therapies to patients with diseases that currently have few therapeutic options

*ACGME* Accreditation Council for Continuing Medical Education, *COI* conflict of interest, *IRB* Institutional Review Board

### Unfunded, investigator-initiated clinical research

**Fig. 3:**
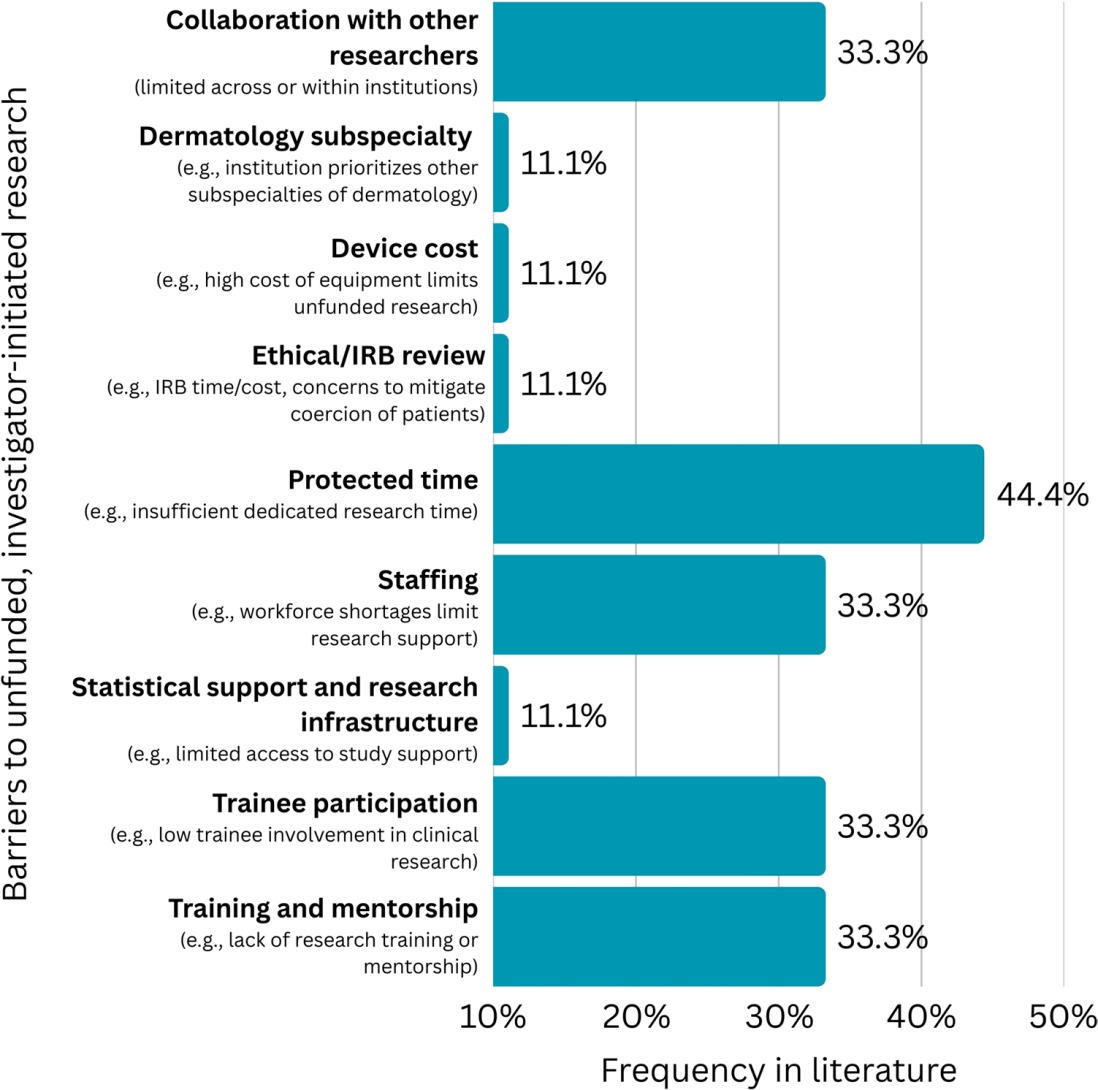
Frequency of barriers to conducting unfunded, investigator-initiated clinical research in cosmetic and laser dermatology in the academic setting, displayed as percentage of total identified articles pertaining to unfunded, investigator-initiated research (N = 9)

Nine barriers to unfunded, investigator-initiated clinical research in cosmetic and laser dermatology in the academic setting were identified, with 20 total possible approaches to overcoming these barriers proposed by the expert panel (Figure 3, Table 4). The most commonly cited barrier was the amount of protected academic time, followed by limited staffing, collaboration with other researchers, training and mentorship, and trainee participation.

**Table 4:** Barriers to conducting unfunded, investigator-initiated clinical research in cosmetic and laser dermatology in the academic setting, including description and proposed possible approaches to overcoming each barrier

Barrier	Description	Possible approaches to overcome each barrier
Collaboration with other researchers	Lack of collaboration with other institutions or investigators within the same institution, both within dermatology and with other specialties, possibly due to low awareness of this option or lack of interest from colleagues [7, 10]	• Medical professional societies can create databases on ongoing investigator initiated studies that are seeking additional sites • Having a single site serve as the coordinating and training site to mitigate the burden on other participating sites • Approaching junior faculty, who may particularly benefit from collaborative research with senior investigators, and who may find that involvement supports their plan for promotion
Dermatology subspecialty	Institutions may place greater priority on different areas of dermatology than the investigator’s particular research interests, or may lack understanding of the relevance of research to the broader population [17]	• Consider partnering with divisions or departments that have similar priorities and goals, or who care for overlapping patient populations • Seek out individuals within the institution who treat the particular condition being studied or who have a particular interest in participating
Device cost	Procurement and maintenance of costly research equipment, such as energy-based devices, may preclude unfunded research [8]	• Enlist interested patients and patient advocacy groups in supporting your research • See if some other division, department, or program within your institution already owns or uses the device in question • Ask the manufacturer for a brief device loan while you conduct the study
Ethical/IRB review	IRB related barriers may include numerous specific issues, including the time and cost of IRB approval, and IRBs recommendations to mitigate the risk of patient coercion [5]	• Administrative support from peers and professional societies [5]
Protected time	Limited protected academic time [9, 10, 15, 18]	• Ask for more protected time and justify this with your research plan
Staffing	Support from office and clinical staff is limited by workforce shortages, and this is amplified in laser and device research, which requires additional safety training [8, 9, 18]	• Involve medical students and residents in supporting your research • Hire a research fellow to support operation of clinical trials • Identify nurses or technicians on the clinical side who may be particularly interested in research and may be willing to help
Statistical support and research infrastructure	Lack of easy access in some cases to biostatistical and study design support [10]	• Use the institutional or departmental biostatics core • Work with students in the mathematics or biostatistics department • Collaborate with other institutions that may have more robust research infrastructure • Enroll in an MPH or other master’s degree program in clinical study design, clinical research, or biostatistics.
Trainee participation	Limited participation by trainees (e.g., fellows, residents, students) in clinical research, possibly due to lack of research mandates or low interest [7, 11, 18]	• Require trainee participate in research [7, 8] • Make trainee participation worthwhile (e.g., write strong letters of recommendation, dedicate time to mentorship, etc.)
Training and mentorship	Lack of training in clinical research methods and adequate research mentors [10, 11, 15]	• Mentorship of trainees and young faculty [10]

*IRB* Institutional Review Board

### Publicly funded, investigator-initiated clinical research

**Fig. 4:**
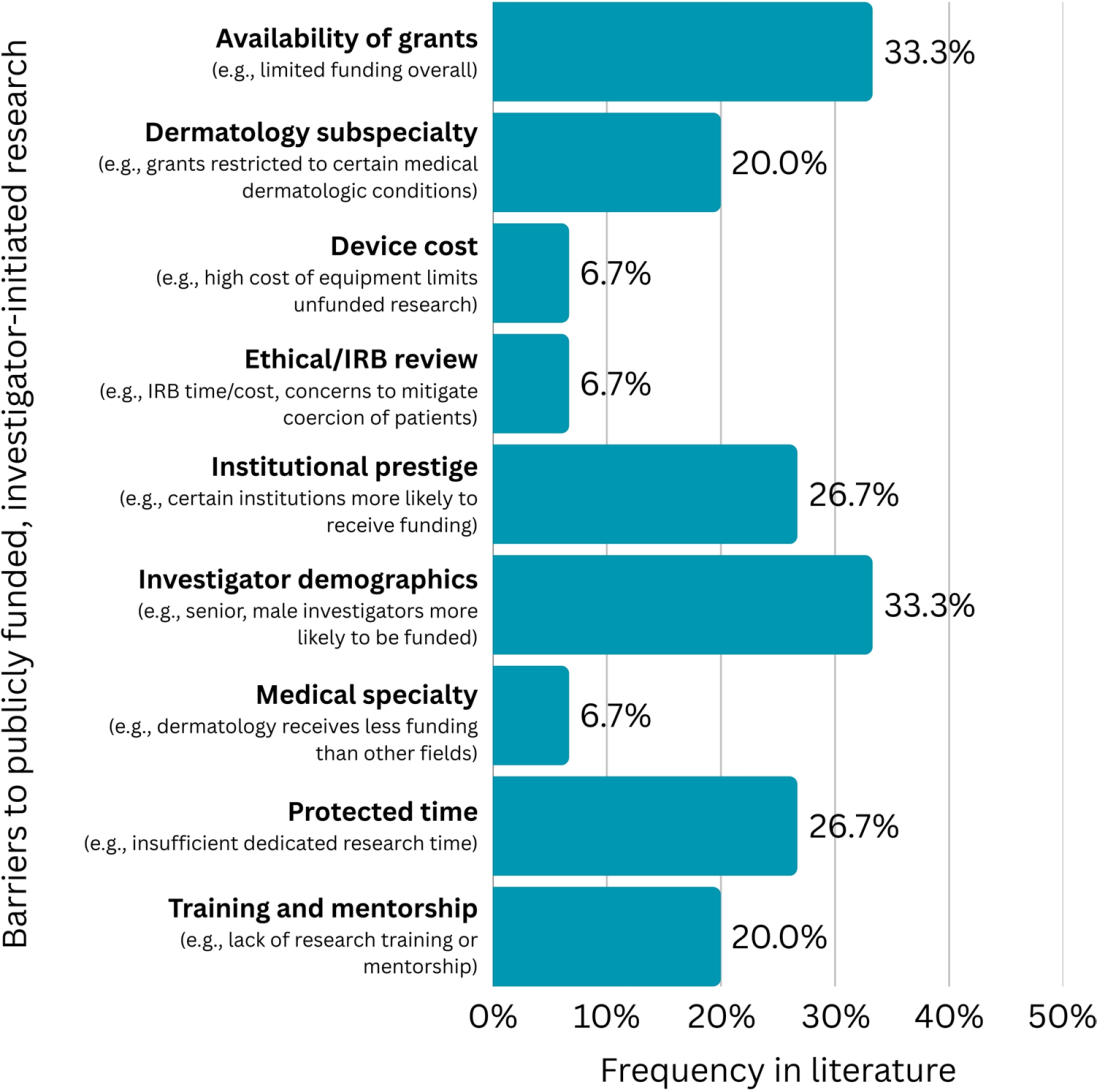
Frequency of barriers to conducting publicly funded, investigator-initiated clinical research in cosmetic and laser dermatology in the academic setting, displayed as percentage of total identified articles pertaining to publicly funded, investigator-initiated research (N = 9)

Nine barriers to publicly funded, investigator-initiated clinical research in cosmetic and laser dermatology in the academic setting were identified, with limited availability of grants and disproportionate awarding of grants based on investigator demographics (e.g., investigators who are men, have a PhD, are of higher academic ranking, etc.) being the most frequently cited barriers (Figure 4, Table 5). Twenty possible approaches to overcoming each barrier were proposed.

**Table 5:** Barriers to conducting publicly funded, investigator-initiated clinical research in cosmetic and laser dermatology in the academic setting, including description and proposed possible approaches to overcoming each barrier

Barrier	Description	Possible approaches to overcome each barrier
Availability of grants	Limited grant funding available [4, 6–8, 10, 11]	• Improve awareness of grant opportunities available from dermatology and disease foundations, professional medical societies, advocacy groups, academic centers, etc. • Leverage advantages of performing trial in academic center (high volume patient base, diverse population, potential for multi-specialty collaboration, ability to perform additional testing: histopathologic, genetic, molecular testing, etc.)
Dermatology subspecialty	Governmental grants are typically awarded for medical dermatologic conditions, and non-government grants may similarly be restricted to specific dermatological conditions, limiting the research questions that can be addressed [8, 13]	• Conduct research on the applications of cosmetic and laser procedures in the treatment of complex medical dermatologic conditions
Device cost	Procurement and maintenance of costly research equipment, such as energy-based devices, may require investment by academic institutions and/or use a significant portion of grant funding [8]	• Enlist interested patients and patient advocacy groups in supporting your research • See if some other division, department, or program within your institution already owns or uses the device in question • Ask the manufacturer for a brief device loan while you conduct the study
Ethical/IRB review	IRB related barriers may include numerous specific issues, including the time and cost of IRB approval, and IRBs recommendations to mitigate the risk of patient coercion [5]	• Administrative support from peers and professional societies
Institutional prestige	More prestigious institutions are more likely to receive funding [6, 12–14]	• Collaborate with other institutions • Develop or join a research consortium • Consider internal grants that may be available from your institution.
Investigator demographics	Investigators who are men, have a PhD, have a higher H-index, are more senior/have a higher academic ranking, and hold leadership positions in professional societies are more likely to receive funding [6, 13, 14, 18, 19]	• Participate in programs for development of minority and female researchers • Reach out for guidance and mentorship from senior investigators who are demographically similar to you and may work at the same or other institutions
Medical specialty	Less funding is available for dermatology than other medical specialties, and this is disproportionate to the burden of skin disease [6, 10, 15]	• Formal collaboration with other medical specialties [15]
Protected time	Limited protected academic time, which is necessary both to secure funding and conduct research [9, 10, 15, 18]	• Ask for more protected time and justify this with your research plan
Training and mentorship	Lack of training in clinical research methods and adequate research mentors [10, 11]	• Mentorship of trainees and young faculty [10]

*IRB* Institutional Review Board

### Limitations

Limitations of this study include that most of the literature reviewed by the expert panel on barriers to funding and conducting research in the academic and private practice settings was not directly related to cosmetic and laser research. In addition, individual research sites, whether in academics or in private practice, may differ with regard to the specific barriers that pose the greatest obstacle to research.

## Conclusion

Conducting cosmetic and laser dermatology clinical research presents distinct challenges in academic versus private practice settings. In academia, barriers such as stringent institutional protocols, a slow and cumbersome process for reviewing ethical considerations, and a bureaucratic approval process can impede the swift initiation of research studies. Funding barriers loom large, as academic researchers often rely on competitive grants, which are also sought by many other peer institutions. Conversely, investigators in private practice can navigate research more expeditiously since there are fewer bureaucratic obstacles, but they also encounter financial constraints. Simultaneously pursuing rigorous scientific inquiry and financial viability remains elusive. Addressing this challenge necessitates collaborative efforts, innovative funding models, and a shared commitment to advancing cosmetic and laser medicine within both academic and private realms.

There are numerous benefits to conducting cosmetic and laser research in the academic setting, despite the obstacles. By fostering creativity and collaborating with a diverse team, including physicians, physician extenders, nurses, scientists, medical students, residents, fellows, biostatisticians, and various professionals, the process of securing funding and conducting clinical research may become more readily attainable and sustainable.

## Data Availability

Data sets generated during the current study are available from the corresponding author on reasonable request.

## Footnotes

1 Saranya P Wyles, MD, PhD; Yakir Levin, MD, PhD; Rawaa Almukhtar, MD; Stephanie R Jackson Cullison, MD, PhD, MS; Jayne S Joo, MD; Sami K Saikaly, MD; Maria Hordinsky, MD; Edit B Olasz Harken, MD, PhD; Diana Bolotin, MD, PhD; Kira Minkis, MD, PhD; Murad Alam, MD, MBA, MSCI.
